# The extent and structure of pig rearing system in urban and peri-urban areas of Guwahati

**DOI:** 10.1080/20008686.2020.1711576

**Published:** 2020-01-08

**Authors:** Sidhartha S. Mohakud, Razibuddin Ahmed Hazarika, Sarat Sonowal, Durlav P. Bora, Archana Talukdar, Santanu Tamuly, Johanna F. Lindahl

**Affiliations:** a Department of Veterinary Public Health, Assam Agricultural University, Guwahati, India; b Department of Veterinary Microbiology, Assam Agricultural University, Guwahati, India; c Department of Veterinary Biochemistry, Assam Agricultural University, Guwahati, India; d Department of Biosciences, International Livestock Research Institute, Nairobi, Kenya

**Keywords:** Pig rearing, urban livestock keeping, urban and peri-urban agriculture, pig husbandry, free-ranging livestock

## Abstract

Livestock is common in Indian cities and contribute to food security as well as livelihoods. Urban livestock keeping has been neglected, and in India, little is known about the topic. Therefore, urban and peri-urban pig farms of Guwahati, Assam, India, were surveyed in order to understand more about the pig rearing systems and risks of diseases. A total of 34 urban and 66 peri-urbanpig farms were selected randomly. All reared cross-bred pigs. Free-range pig rearing was common in both urban (58.8%) and peri-urban (45.45%) farms. Artificial insemination was used by around half of the pig farmers. Disinfection in pig farms was practiced in 26.5% of urban and 28.8% of peri-urban farms. More urban pig farms were observed to be moderately clean in (82.4%) compared to peri-urban (69.7%). However, more urban (67.7%) than peri-urban farms (57.6%) reported ahighrodent burden. Pig sheds were mostly basic, with bricked floors in 18.2% farms in peri-urban areas, and more than 80% had corrugated iron roofing sheets. In conclusion, free-roaming pigs in both urban and peri-urban areas of Guwahati can contribute to disease transmission, and the low standard of hygiene and buildings may further increase the risk of diseases.

## Introduction

The livestock sector plays a crucial role in the rural economy and livelihood, but with growing urban populations the livestock sector is increasing both in cities and in the surrounding areas. In India, this sector contributes 4.1% GDP and 25.6% of the total agriculture GDP, and the country comprises 11.6% of world livestock population [1,2]. Livestock has become a mainstay in income, employment, food, social security, draught, and manure. The livestock sector employs 8% of the country’s labor force, including many small and marginal farmers, women, and landless agricultural workers in the sectors such as the milk production, organic fertilizer, and important input to crop production [2].

Among the livestock species, pigs find an important place as they are being reared by socio-economically weaker sections of the society. According to the 19^th^ livestock census of India, pigs comprise 2.0% to the total livestock population and about 10 million pigs which contribute about 6.7% of the total meat production in the country [3]. Pigs, as compared to larger livestock species, have a great potential to contribute to faster economic return to the farmers, because of certain inherent traits like high fecundity, better-feed conversion efficiency, early maturity, and short generation interval [4]. The pig requires minimum capital investment, labor, buildings, and equipment, and can easily be kept in cities [5]. The pig farming sector is highly un-organized in most parts of India as the pig population is reared under traditional smallholder, low-input, demand-driven production systems. The distribution of the pig population across the country is not uniform with the highest population of pigs in the eastern and north-eastern states. The highest population is in Assam (2 million), followed by Uttar Pradesh (1.4 million), West Bengal (0.8 million), Jharkhand (0.7 million), and Nagaland (0.7 million) [3].

Assam is a state in Northeastern India and its economy is mainly dependent on agriculture and allied activities. Livestock is reared by almost every household in the rural areas of Assam. Non-descript cattle, small ruminants, mainly goats, and backyard poultry are common along with agriculture, while pig rearing is mostly done in areas dominated by scheduled tribes and scheduled castes, historically disadvantaged groups in India [6,7]. Pig farming in Assam has established itself as a major livelihood supporter for poor, marginalized, and landless farmers. The pig sector in the state of Assam has major challenges such as shortage of feed and feed crops, low productivity of indigenous pigs, infectious and metabolic diseases and impact of climate change and global warming, which need to be addressed enabling the sector to grow according to its potential [8]. A large number of infectious diseases that are prevalent in Assam have serious implications on pig productivity, export potential, safety, and quality of pig products. Many zoonotic diseases are present in Assam, including brucellosis, leptospirosis, hydatidosis, cysticercosis, taeniasis, and toxoplasmosis [9–12]. These zoonotic diseases can have a great impact on the livelihood of livestock farmers by affecting their health and reducing the quantity and quality of animal products. Particularly in an urban setting, where livestock and humans live more crowded, keeping animals such as pigs may increase the risk of zoonotic infections in humans [13,14]. Urban livestock systems have been little studied in India, and particularly not in the poorer Northeast region where Assam is located. The present study was carried out to understand the urban and peri-urban pig rearing systems in Guwahati, Assam, and its social and economic implications, as well as its sustainable livelihood possibilities.

## Materials and methods

### Ethical approval

This study was approved by the Institutional Animal Ethics Committee vide approval No.770/ac/CPCSEA/FVSc/AAU/IAEC/17-18/590 dated 09.08.2017, Assam Agricultural University, Khanapara, Guwahati. Farmers were well informed about the intent and purpose of this study and were only interviewed after they had given their consent.

### Farm selection

Urban farms were defined as within the official city boundaries. Peri-urban was defined as within 10 km of the official city boundaries of Guwahati, the capital of Assam, and all villages in that circle were mapped and pig farms identified. For the purpose of this study, 100 farms (34 from the urban area and 66 from the peri-urban zone) with a herd size of 3 or more adult pigs were selected randomly. Locations were properly recorded using GPS tools.

### Data collection

The collection of information was done between July 2017 and June 2018. A questionnaire was prepared and data were collected through on-site interviews in the local Assamese language, with one of the authors visiting each farm and asking all questions to the farmer. Observations about cleaning practices and hygienic status of farms were made using an observation checklist.

## Data analysis

Data were entered into Microsoft Excel® for office 365 and was analyzed statistically using SAS 9.3 software and STATA 14.2. Averages are presented with standard deviation (SD).

## Results

### Baseline information about basic demography and farm details of urban and peri-urban pig farmers

In total 65 women and 35 men answered the questionnaire. The number of pigs (including piglets and fatteners) kept by female (average 6.9, SD 2.0) or male (6.2, SD 2.3) farmers or between peri-urban and urban farms (Table 1), were not significantly different.

**Table 1.** The number of pigs kept on urban and peri-urban farms in Guwahati, Assam, India

	Total pigs kept*				Sows				Boars			
	Mean	SD	Min	Max	Mean	SD	Min	Max	Mean	SD	Min	Max
Peri-urban	6.7	2.4	3	15	2.2	1.2	0	5	0.2	0.4	0	1
Urban	6.6	1.6	3	9	2.4	0.8	1	4	0.4	0.5	0	1
Total	**6.6**	**2.2**	**3**	**15**	**2.3**	**1**	**0**	**5**	**0.3**	**0.5**	**0**	**1**

SD = Standard deviation, Min = minimum, Max = maximum *Total pigs include sows, boars, piglets and fattening pigs

The majority of the pig farmers in urban (58.8%) and peri-urban (57.6%) areas had obtained their secondary level of education but many farmers (17.7% of urban farmers and 13.64% of peri-urban) completely lacked education. While there was no difference between urban and peri-urban areas, there were significant differences between females and males (p < 0.001), with 40.0% of male respondents lacking education (not having attended schools, analphabets), compared to 1.5% of the female, while 73.9% of women had education up to class 10, and only 28.6% of the mean. All the farmers reared cross-bred pigs. In urban areas, 85.3% of the farms procured new stocks from unknown sources or markets, as compared to 66.7% farms in peri-urban areas.

In most of the pig farms of both urban (82.4%) and peri-urban (87.9%) areas, no quarantine procedure was followed when introducing new pigs to the farm. Interestingly, female respondents were less likely to report to have a quarantine than men (6.2% and 28.6%, respectively, p = 0.002). The free-range, scavenging, pig rearing system was recorded to be the most commonly adopted rearing system in 58.8% urban and 45.45% peri-urban areas of Guwahati followed by semi-intensive (outside pen) and intensive system (indoors). Regarding breeding methods followed by pig farmers, artificial insemination was used by about half of the farmers (52.94%) in urban areas of Guwahati. More urban (67.7%) pig farms than peri-urban (57.6%) pig farms of Guwahati reported rodents as a big nuisance. Most of the pig farms, both in urban (82.4%) and peri-urban (83.3%) areas of Guwahati, were found to be devoid of wild animal contact, and less than half of the pigs were reported to have contact with other domestic animals (48.5% and 47.1% in peri-urban and urban farms, respectively). Dogs were recorded to be the predominant in-contact animal in both urban and peri-urban farms. Concrete floors were recorded in 76.5% and 72.7% of the pig farms in urban and peri-urban areas, respectively. Most of the farms in urban (82.4%) and peri-urban (83.3%) areas of Guwahati recorded the use of corrugated iron roofing sheets.

### Hygiene standards and disease prevention

Use of disinfection was reported in 26.5% and 28.8% urban and peri-urban farms, respectively. In aspect of cleanliness, 82.4% of the pig farms in urban areas were judged moderately clean as compared to 69.7% in peri-urban areas and no dirty farm was recorded in urban areas in contrast to 7.6% dirty pig farms in peri-urban areas of Guwahati. Vaccination was done by less than half of the farms under study, 47.1% of urban and 48.5% of peri-urban farms. The diseases for which animals were vaccinated in urban and peri-urban pig farms included classical swine fever, haemorrhagic septicemia, and foot and mouth disease.

## Discussion

This study aimed at understanding more about the urban and peri-urban pig keeping that is often neglected from an extension and policy standpoint and that can significantly contribute to increased exposure of zoonotic diseases in humans [13,15]. The question on educational status of pig keepers in the study area revealed that most of the pig farmers were educated up to the secondary level of education in Guwahati. This study is in accordance with the report by Patr et al. [16], where 44% of pig farmers had education below class 10. Cross-bred pigs were most common in this study, which has also been found to be among the pig farmers in Nagaland, a neighbouring state [16], motivated by better growth performance, higher weight gain, larger litter size, greater back fat thickness [17].

In this study, most of the pigs acquired to the farms in urban as well as peri-urban areas of Guwahati were from unknown sources. Pigs from unknown herds of unknown health status always pose a risk of harbouring diseases compared to those procured from known sources such as government farms [18]. In most of the pig farms of both urban and peri-urban areas of Guwahati, quarantine procedures were not followed. This is not only bad in relation to the risk of spreading diseases, and according to FAO [18], the adjustment of newly arrived pigs is crucial to the conditions prevailing in the farm enabling them to optimal performance. During the present study, free-range pig farming was found to be the most commonly adopted rearing system in urban and peri-urban areas of Guwahati followed by semi-intensive and intensive system. Ahmed et al. [19], however, reported that pigs were reared under neck/girth tethering (83%) followed by straw-shed house (12%), fencing system (4%), and penned system (1%) in Assam, respectively. Our results show that artificial insemination was followed by most of the pig farmers in urban and peri-urban areas of Guwahati. This is in agreement with the findings of Rahman et al. [20], where he found that most of the farmers in northeastern states applied artificial insemination in pig farms. Artificial insemination has been promoted and led by the National Research Centre on pigs, a part of the Indian Council of Agricultural Research [21]

The present study revealed that disinfection was not practiced in most of the farms in both urban and peri-urban areas of Guwahati. The preference for concrete floor by the pig farmers in both urban and peri-urban areas of Guwahati in the present study might be due to its strength, durability, as well as easy cleaning and drainage facilities. On the other hand, it was earlier reported [22,23] that the pigsties were made of either concrete (36.5%) or katcha (63.5%) floor in peri-urban areas like Kamrup and Darrang districts of Assam, which may indicate that the hygienic standards have been improving. Similarly, in the present study, most (>80%) of the farms in urban and peri-urban areas of Guwahati used corrugated iron roofing sheets, while earlier studies found that the roof of pigsties in Assam was made up of thatch (49.3%) with locally available straw and coconut leaf followed by plastic cover (28.6%) [24].

Around half the farms in both urban and peri-urban areas of Guwahati let the pigs get in contact with other species, of which dogs were the most frequently reported contact species. In the present investigation, the nuisance of rodents was found to be comparatively higher in urban pig farms than peri-urban pig farms of Guwahati. Rodents cause immense losses due to destruction and contamination of food, and also transmit several potentially fatal diseases, including leptospirosis, to both humans and animals [25]. Low hygienic standards of the pigsties can also contribute to breeding grounds of mosquitoes, and it has been shown in Vietnam that urban households with pigs are more exposed to vectors of Japanese encephalitis virus [14,26], a virus that is frequently causing human disease in Assam [9].

## Conclusion

This study emphasizes the need to scaling up the level of education for effective pig farming to increase the income of the farmers of urban and peri-urban areas of Guwahati belonging to lower socio-economic strata. Lack of awareness among pig farmers on the selection of new animals is the need for targeted for maintenance of a healthy stock. The unhygienic pig rearing practices currently followed by pig farmers necessitate proper guidance from the scientific community and involvement of various stakeholders to protect them from various zoonotic diseases through the implementation of proper managemental practices with high standards of hygiene. The need for adequate use of disinfectants is required for maintaining hygiene in urban and peri-urban pig farms.
